# The effect of endpoint congruency on bimanual transport and rotation tasks

**DOI:** 10.3389/fpsyg.2015.00092

**Published:** 2015-02-10

**Authors:** Andrea H. Mason, Pamela J. Bryden

**Affiliations:** ^1^Department of Kinesiology, University of Wisconsin–Madison, Madison, WIUSA; ^2^Department of Kinesiology and Physical Education, Wilfrid Laurier University, Waterloo, ONCanada

**Keywords:** bimanual movements, movement synchrony, motor planning, movement constraints, endpoint congruency

## Abstract

The completion of many goal oriented skills requires the tight coordination of the right and left hands to achieve the task objective. Although the coordination of wrist transport and orientation of the hand before object contact has been studied in detail for discrete bimanual tasks, as yet, very few studies have examined bimanual coordination when the target is already in hand. It has been shown that congruency of the goal facilitates the production of discrete bimanual responses. The purpose of this study was to investigate the role of goal congruency on precision bimanual transport and rotate tasks. In the current investigation, participants transported two cubic objects while rotating them laterally to place them into tight-fitting targets. The magnitude of the rotation could be the same for both hands (i.e., both 45 or 90^∘^) or different (i.e., one 45 and 90^∘^) and the endpoint orientations (i.e., goal) could either be congruent or incongruent. Results indicated that when the endpoint orientation was congruent for the two hands, movement times were similar regardless of hand (left or right), rotation magnitude (45, 90^∘^) and whether the rotation magnitude for the two hands was the same or different. These results suggest that congruency of the endpoint goal facilitates the temporal synchronization of the transport component for two limbs. In contrast, a different pattern of results was obtained when considering the rotation component. Specifically, regardless of whether the hands were rotating the same magnitude or ending in congruent endpoint positions, the coordination of the rotation component between the hands was asynchronous. We hypothesize that the greater requirement to shift visual fixation from one hand/target to the other to ascertain the separate goal orientations may explain these differences. These results provide further evidence that multiple constraints act to influence the performance of skilled bimanual tasks.

## INTRODUCTION

The performance of many goal oriented skills requires the tight coordination of the right and left hands to achieve the task objective. Consider tying your shoelaces or opening the cupboard door with your right hand while grasping a cup with your left hand. These tasks require precise spatial and temporal coordination between the two hands for the goal of the task to be successfully achieved.

Bimanual performance has received much attention in recent years, with several studies investigating how movements are planned based on direct or indirect spatial cues (Diedrichsen et al., 2003), how they are temporally and spatially coupled (Kelso et al., 1979; Franz et al., 1991, 2001; Dohle et al., 2000) and how movements are altered based on visual feedback (Bingham et al., 2008; Mason, 2008; Srinivasan and Martin, 2012). Recently Srinivasan and Martin (2012) have shown that with practice on a bimanual reaching task, participants begin to prioritize one hand over the other. Their results indicated that for their group of participants, the left hand became the primary hand, with gaze biased in that direction. Further, left-hand kinematics remained similar in unimanual and bimanual trials, while right-hand kinematics varied with task constraints. Although these studies have provided important descriptions of bimanual performance, they have focused almost exclusively on the planning and performance of movements prior to object contact. For most functional tasks, object manipulation does not end when the object is acquired, therefore a thorough investigation of coordination during the object manipulation phase of the movement is required.

In a previous series of studies, we investigated the coordination and concurrency of bimanual movements made by participants to simultaneously transport, rotate and place two objects into target wells (Mason and Bryden, 2007). The target wells were oriented such that participants had to rotate the objects 45 or 90^∘^ to achieve the task goal. Results indicated that the two hands were tightly synchronized when the two movements being performed required the same rotation. Specifically, transport and rotation movements for the two hands started and ended at the same time. However, when participants performed bimanual movements where the rotations were different, synchronization between the two hands was weaker and was influenced by the type of rotation being performed by each hand. The hand rotating to a 45^∘^ target ended the transport component later and the rotation component earlier than the hand moving to the 90^∘^ target. Further, the hand performing the 45^∘^ rotation committed a larger number of over-rotations than the hand performing the 90^∘^ rotation, resulting in less efficiency in the movement when compared to the unimanual conditions. These results suggest that movement symmetry acts as a constraint to significantly influence the planning and performance of bimanual skills.

Another constraint that has recently been shown to significantly influence and facilitate the production of discrete bimanual responses is the congruency of the endpoint goal (Kunde and Weigelt, 2005; Kunde et al., 2009; Hughes et al., 2011). Using a task inspired by Rosenbaum et al. (1990), Kunde and Weigelt (2005) investigated whether goal symmetry or movement symmetry has a greater influence on bimanual task performance. They manipulated goal congruency by asking participants to place objects in either parallel (i.e., both upright or both upside down) or opposite (i.e., one upright, one upside down) orientations. These goals could be achieved by either mirror-symmetrical (i.e., both hands turning inward or outward) or mirror-asymmetrical (i.e., one hand turns inward, one hand turns outward) movements. The authors suggested that if movement symmetry dominates the planning and performance of bimanual movements, performance would be better for mirror-symmetric movements regardless of endpoint goal congruency. In contrast, if endpoint goal was more important, then better performance would be exhibited with congruent endpoint goals regardless of movement symmetry. Their results indicated that reaction times, approach times, and manipulation times were strongly influenced by goal congruency but were not significantly influenced by movement symmetry. This led the authors to conclude that goal congruency (i.e., the “what” of actions) is crucial to motor planning and performance whereas the motor patterns used to achieve these goals (i.e., the “how” of actions) is less important. While the dominance of goal congruency over movement symmetry has been replicated (e.g., Weigelt et al., 2006), other studies have found mixed results. Specifically, Janssen et al. (2009) found the result, but only for the right hand. Others have reported that there is no preference for end-state planning over movement symmetry (Fischman et al., 2003; Hughes and Franz, 2008; Huhn et al., 2014). These conflicting results have led researchers to suggest that multiple planning constraints interact to allow flexibility in motor behavior in a dynamic and task dependent manner (van der Wel and Rosenbaum, 2010; Huhn et al., 2014).

In our previous studies (Mason and Bryden, 2007) the grasped targets always had spatially congruent start positions. This meant that asymmetric bimanual rotations also resulted in incongruent goal positions. Therefore, rotation magnitude (i.e., movement symmetry) and endpoint congruency were confounded. As such, we were not able to determine whether goal congruency plays a role in movement planning and execution for our task. Our task differs from those used by others studying constraints in movement planning in two respects. First, our task required both the transport of a grasped object toward a target location as well as the rotation of the object to place it in a target well. It is possible that each component of the movement (transport vs. rotation) might be influenced differentially by task constraints. This notion follows from work in reach-to-grasp movements where it has been shown that certain environmental constraints influence the transport but not the grasp or vice versa in a task dependent way (Gentilucci et al., 1991; Carnahan and McFadyen, 1996). The second difference in our paradigm when compared to previous work is the increased precision requirement inherent in the final goal. Specifically, in previous works, participants either rotated dowels to place them with a specific end facing upward or grasped plungers to move them to higher or lower shelves (Fischman et al., 2003; Kunde and Weigelt, 2005; Hughes and Franz, 2008; Huhn et al., 2014). In these paradigms, the precision required to successfully place the object at the end location was relatively low. In contrast, in our paradigm, participants need to precisely rotate the object in order to fit it into a tight target well. Thus, the increased precision requirements in both the movement and the end-goal introduce an additional constraint on the task that could supersede other constraints.

The purpose of this study was to investigate how movement and goal congruency interact to influence the transport and grasp components of a grasp and place task when precision requirements are high. By manipulating the congruency of the starting orientations, the endpoints (i.e., goal) could be congruent or incongruent for a given set of rotations. With these manipulations, we could determine whether decreases in movement synchrony are still observed in asymmetric conditions regardless of goal congruency. This result would suggest that precision requirements reduce the beneficial effects of goal congruency. In contrast, if movement synchrony was observed in asymmetric rotation conditions for the congruent endpoints, this would suggest that goal congruency is an important planning variable regardless of precision requirements.

## MATERIALS AND METHODS

### PARTICIPANTS

Twelve participants (six female, six male) with a mean age of 21.4 (range: 20–27) years participated in this study. All participants were right-hand dominant as assessed by the Waterloo Handedness Questionnaire (Bryden, 1977). Ethical approval from the University of Wisconsin–Madison Social and Behavioral Sciences Institutional Review Board and the Research Ethics Board at Wilfrid Laurier University was obtained before testing began. Participants had no prior knowledge of the experiment and were asked to provide informed consent before beginning the study. Each participant performed in one experimental session for approximately one half hour.

### APPARATUS

**FIGURE 1:**
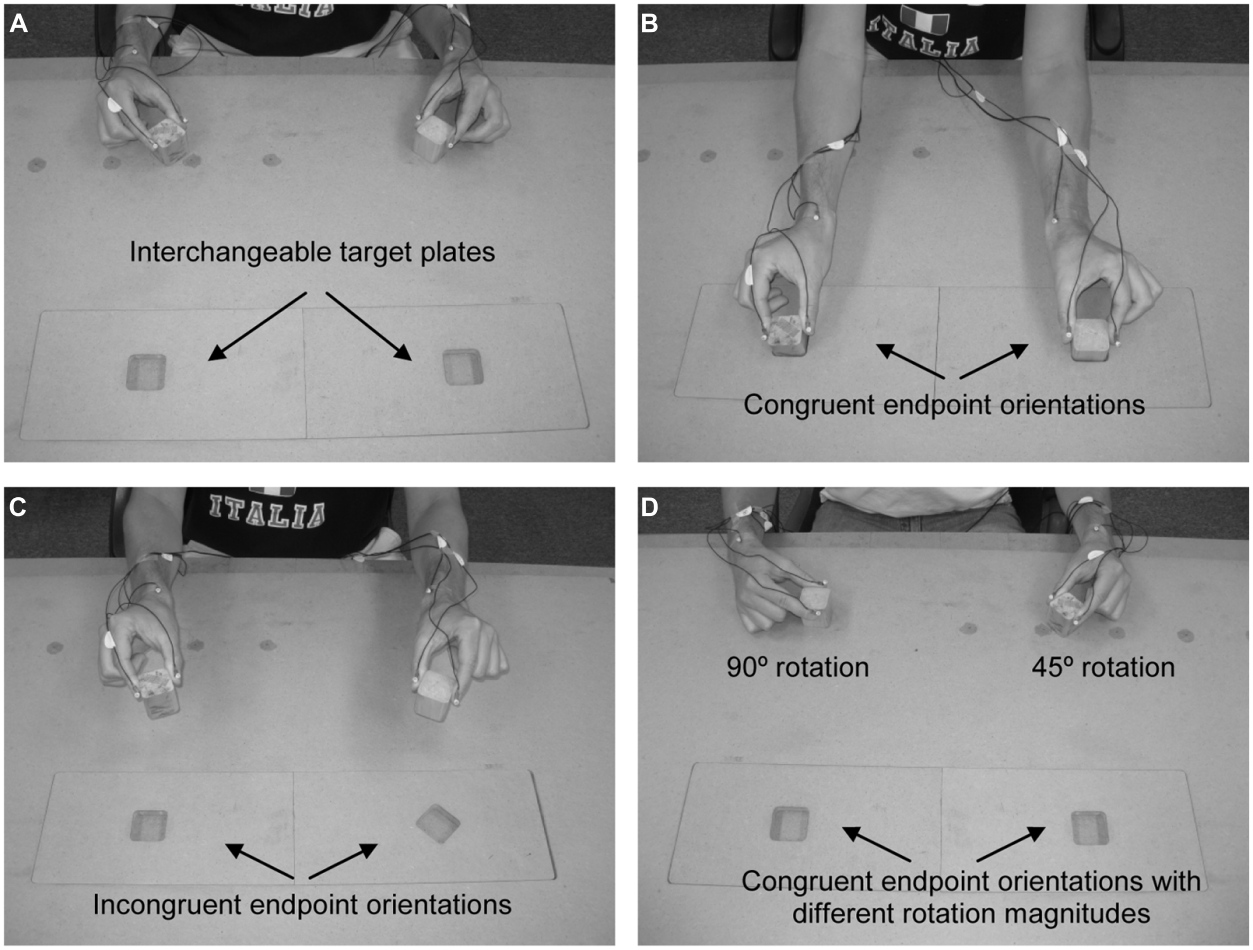
**General layout of experimental setup.** Target plates were interchangeable, allowing for congruent and incongruent endpoint orientations when participants rotated the cubes either 45 or 90^∘^. **(A)** shows the interchangeable target plates, **(B)** shows the position of the hands and cubes for congruent endpoint orientations, **(C)** shows target plates that require incongruent endpoint orientations of the hands and cubes, and **(D)** shows different start positions for the hands and cubes, which lead to congruent endpoint orientations despite different rotation magnitudes.

Participants were seated facing a table on which a 48 cm × 96 cm sheet of medium density fiberboard (MDF) was fastened. A 15 cm × 59 cm rectangle was cut out of the sheet of MDF such that interchangeable target plates could be positioned in the rectangular cutout (see **Figure 1A**).

Kinematic data were recorded for the participants’ hand movements using a VisualEyez 3000 (Phoenix Technologies Inc.) three-dimensional motion capture system. The VisualEyez camera monitored the position of light emitting diodes (LEDs) placed on both hands in the following locations: thumb – dorsoradial aspect of the distal phalanx, index finger – dorsomedial aspect of the distal phalanx, wrist – radial aspect of the distal styloid process. LEDs were also positioned on both cubic wooden objects, which measured 4 cm × 4 cm × 4 cm. Position data from the LEDs were sampled at 200 Hz, stored, and then analyzed off-line using custom software (KinSys, Eh-Soft).

### PROCEDURE

Before beginning each trial, the height of the participant’s seat was adjusted so that their elbows were flexed at 90^∘^ with both forearms parallel to the floor when their hands were positioned at the start position. No other adaptations for the participant’s body measurements were made (i.e., reach distance and object size was the same for all participants). They grasped one object with the right hand and one object with the left hand using a precision grip. The objects were placed on two start positions located 12 cm to right and left of the participant’s midline. Participants initiated their movements on a verbal “Go” signal provided by the experimenter.

The task was to transport the two objects 30 cm from the start positions while rotating them either 45 or 90^∘^ outward (i.e., laterally) to place them into target wells (**Figures 1A,B**). Outward rotation of the blocks was demonstrated to participants, and they were instructed that all trials required a rotation movement (i.e., even when a rotation of 0^∘^ would allow them to place an object in the target, as shown for the 90^∘^ target in **Figure 1D**, they were asked to rotate the object). Target wells were the same size as the objects, resulting in a tight fit. Participants were asked to move at a comfortable pace and no instructions were given regarding the simultaneity of transport or rotation movements of the right and left hands. All trials were performed with each hand acting on the corresponding side of space (i.e., the right hand moved in right space). Participants were given three practice trials in the congruent condition prior to the beginning of data collection.

The magnitude of the rotation movements required to place the objects within the target wells could be the same (i.e., both 45 or 90^∘^) or different (i.e., one 45 and 90^∘^) for the two hands. Endpoint congruency (i.e., goal) was also manipulated such that the hands ended either in the same orientation (congruent; see **Figure 1B**) or in different orientations (incongruent; see **Figure 1C**). To achieve differences in endpoint congruency for the same rotation magnitude (or alternatively, congruent endpoints with different rotation magnitudes), the orientation of the object at the start position was manipulated (see **Figure 1D**). Any combination of start position and rotation magnitude that caused an outward rotation of the hand past the posture shown in **Figure 1B** was removed. Further, although more than one combination of start orientations could satisfy the incongruent L45R45 and incongruent L90R90 conditions, to maintain a balanced design we chose only one combination. While it is possible that the start orientation may have an asymmetric influence on the two hands, we feel that testing this effect is beyond the scope of the current study. The start and end orientations for both cubes in each condition are represented in **Table 1**. Light pencil outlines of the cubes for each orientation were used by the experimenter to place the object in the starting orientation for a given trial. The participant was then asked to grasp the cube at that starting orientation. The experimenter visually confirmed that the object had not been re-oriented by the participant prior to the start of the trial.

**Table 1:** Starting and ending orientations for the hand/object in each of the eight conditions.

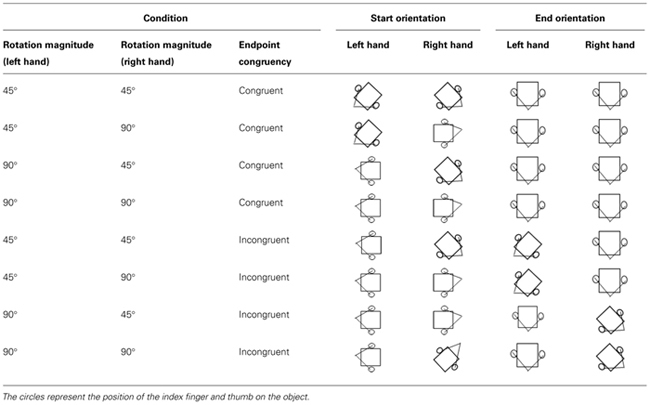

Manipulation of the rotation magnitude (45^∘^, 90^∘^), rotation magnitude congruency (same, different) and endpoint congruency (congruent, incongruent) factors resulted in a total of eight conditions. Each participant completed 10 trials in a blocked order for each of the conditions for a total of 80 trials^1^. The conditions were presented in a random order.

### DATA ANALYSIS

#### Transport and rotate

The three-dimensional position data recorded from the LEDs positioned on the index finger, thumb, and wrist of both hands were first interpolated over missing data points of no more than 20 ms and filtered using a dual-pass second order Butterworth low pass filter with a cutoff frequency of 7 Hz.

**FIGURE 2:**
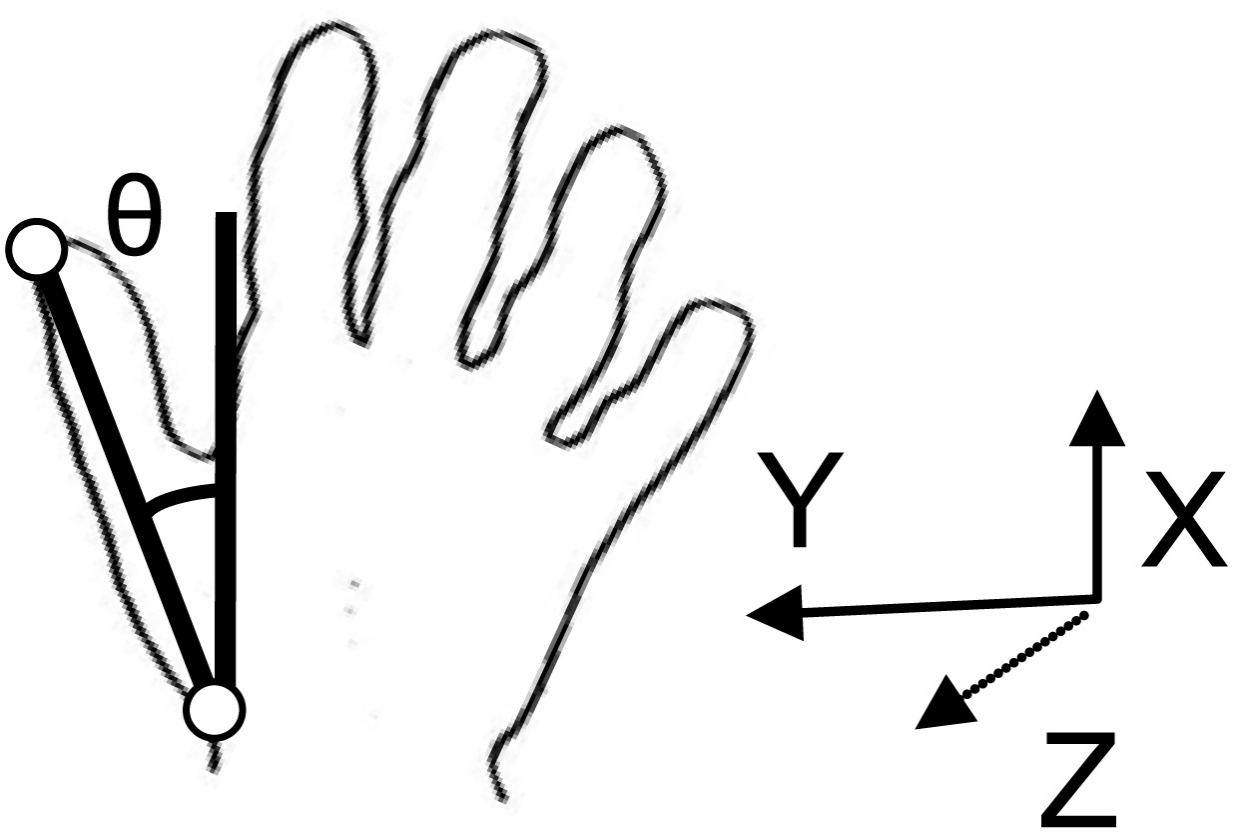
**Rotation of the object was determined using the light emitting diodes (LEDs) on the thumb and wrist.** Rotation was defined as the change in the angle between the *X*-axis and the straight line connecting the LEDs on the wrist and thumb, with the origin passing through the wrist LED.

Movements were divided into two components; object transport toward the target location and rotation of the object to match the orientation of the target well. Start of object transport was defined as the point where tangential wrist velocity increased above a threshold of 5 mm/s and continued to rise. The end of object transport was determined as the time after peak velocity when the wrist velocity in the forward (x) direction first decreased below a threshold value of 5 mm/s. The main kinematic measure of interest for object transport was transport time. Rotation of the object by the hand was determined using the LEDs on the thumb and wrist. Rotation was defined as the change in the angle between the *X*-axis and the straight line connecting the LEDs on the wrist and thumb, with the origin passing through the wrist LED (see **Figure 2**). Note that an angle of 0 was recorded when the line connecting the wrist and thumb was parallel to the *X*-axis, whereas an angle of 90^∘^ was recorded when the line connecting the wrist and thumb was parallel to the *Y*-axis. The start of rotation was defined as the first occurrence of a rotation velocity of greater than 1^∘^ per second. End of rotation was determined as the point after the peak where rotation velocity decreased below a value of 1^∘^ per second. The main kinematic measure of interest for object rotation was rotation time.

Mean values for the 10 trials in each condition for the congruent endpoint orientations were submitted to separate 2 endpoint congruency (congruent, incongruent) × 2 hand (left, right) × 4 condition (L45R45, L90R90, L45R90, L90R45) repeated measures analyses of variance (ANOVA). When significant three-way interactions were found, means were compared separately for the congruent and incongruent endpoints using 2 hand (left, right) × 4 condition (L45R45, L90R90, L45R90, L90R45) repeated measures ANOVA. An *a priori* alpha level of *p* < 0.05 was used to determine significance.

#### Relative difference between right and left hands

To describe the temporal coordination between the movements of the two hands, we calculated relative timing differences between the left and right hands for transport start and end time and rotation start and end time. Negative values indicate that the left hand began/ended before the right hand. Means were submitted to separate 2 endpoint congruency (congruent, incongruent) × 4 condition (L45R45, L90R90, L45R90, L90R45) repeated measures ANOVA. When significant two-way interactions were found, means were compared separately for the congruent and incongruent endpoints using four condition (L45R45, L90R90, L45R90, L90R45) repeated measures ANOVA. An *a priori* alpha level of *p* < 0.05 was used to determine significance.

#### Relative difference between transport and rotate components: concurrency

To examine the temporal concurrency of the transport and rotation components we calculated the relative difference between start of transport and start of rotation (relative transport/rotation start time) and relative difference between end of transport and end of rotation (relative transport/rotation end time). Negative values indicate that the transport component began/ended before the rotation component. These measures were analyzed using separate 2 endpoint congruency × 2 hand (left, right) × 4 condition (L45R45, L90R90, L45R90, L90R45) repeated measures ANOVA. When significant three-way interactions were found, means were compared separately for the congruent and incongruent endpoints using 2 hand (Left, Right) × 4 condition (L45R45, L90R90, L45R90, L90R45) repeated measures ANOVA. An *a priori* alpha level of *p* < 0.05 was used to determine significance.

## RESULTS

To simplify presentation and interpretation of the results, statistics for only the highest order significant interaction are presented and discussed in the text and figures below.

### TRANSPORT AND ROTATE TIMES

**FIGURE 3:**
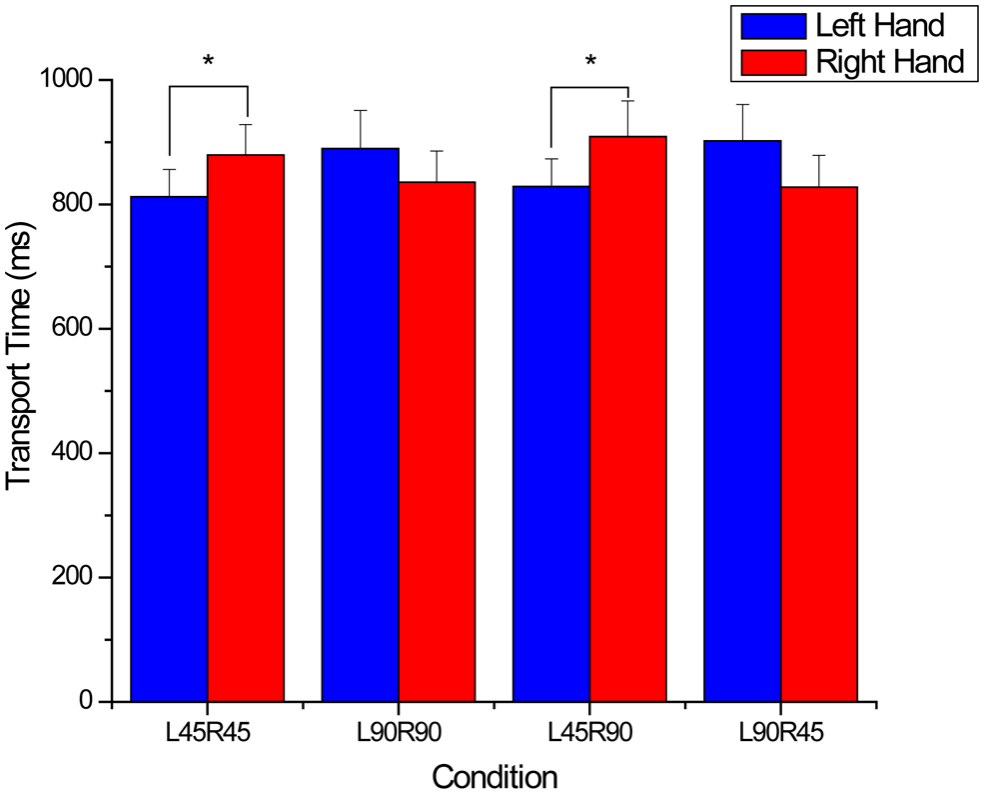
**Transport times for incongruent endpoints.** For the x-axis titles, L and R refer to the left and right hands, and 45 and 90 refer to 45 and 90^∘^ rotations. Note that the left hand was significantly faster when the rotating 45^∘^. *denotes significant (*p* < 0.05) differences between means and error bars represent SE.

For transport time, a significant endpoint × hand × condition interaction was found (*F*_3,33_ = 9.05, *p* < 0.001). The interaction was further decomposed by separately comparing hand and condition within the congruent and incongruent endpoint orientations. For the congruent endpoint orientations, no significant main effects or interactions were found for transport time. Overall participants took 870 ± 47 ms to transport the object from the start position to the target when endpoint orientations were congruent. When the endpoints were incongruent there was a significant interaction between condition × hand (*F*_3,33_ = 15.38, *p* < 0.001; see **Figure 3**). *Post hoc* analysis testing simple main effects of hand within condition revealed that the left hand was significantly faster than the right hand in conditions where the left hand rotated the object 45^∘^ (L45R45: *p* = 0.001; L45R90: *p* = 0.009). Differences between the right and left hands for the other two conditions (L90R90 and L90R45) were not significant.

**FIGURE 4:**
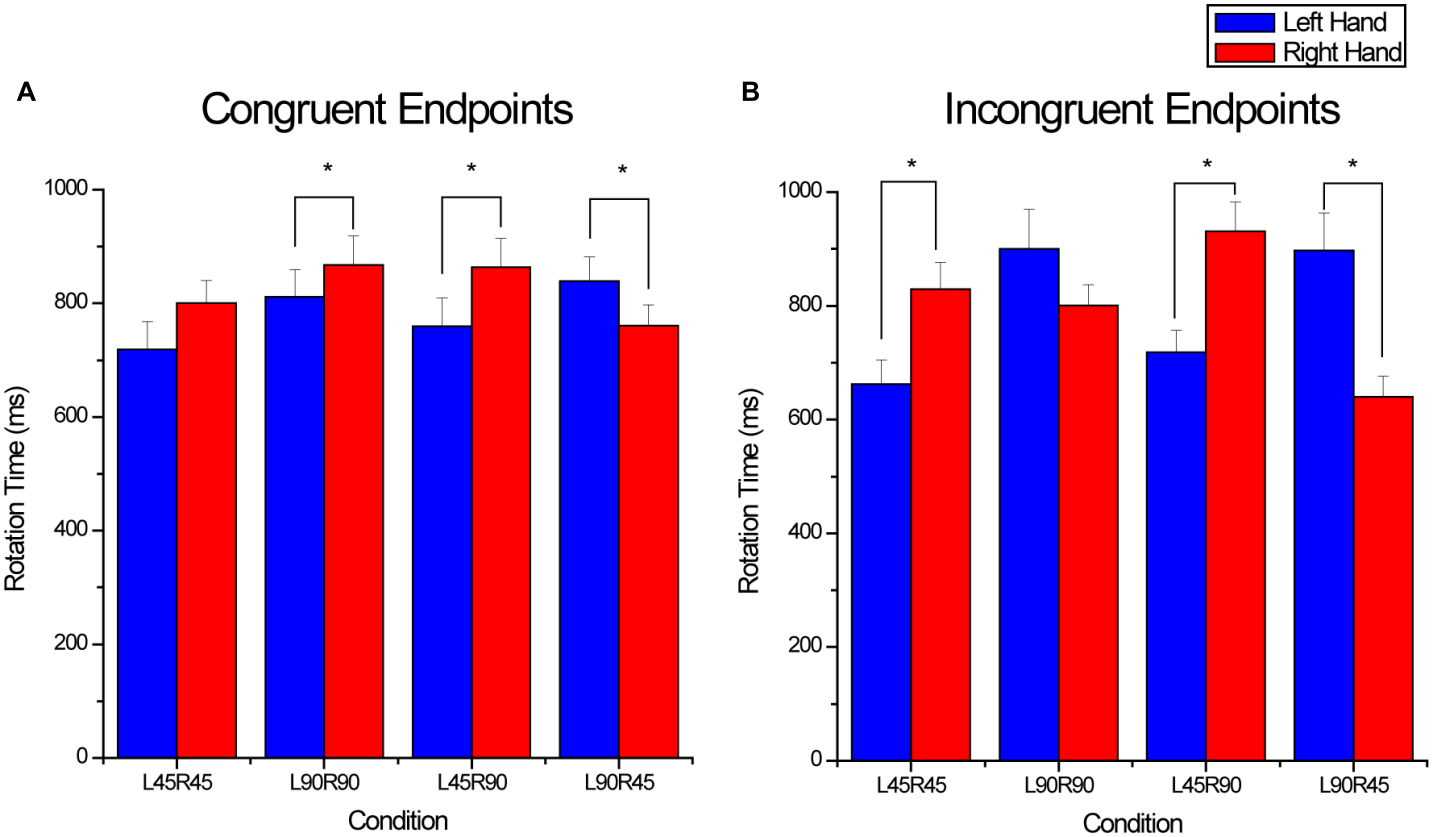
**Rotation times for the **(A)** congruent endpoint orientations and **(B)** incongruent endpoint orientations.** For the x-axis titles, L and R refer to the left and right hands, and 45 and 90 refer to 45 and 90^∘^ rotations. Even when endpoint orientations were congruent and rotation magnitude was similar [e.g., L45R45 and L90R90 conditions in **(A)**], rotation times were significantly different for the two hands. *denotes significant (*p* < 0.05) differences between means and error bars represent SE.

For object rotation time a significant endpoint × hand × condition interaction was found (*F*_3,33_ = 17.7, *p* < 0.001). The interaction was further decomposed by separately comparing hand and condition within the congruent and incongruent endpoint orientations. For the congruent endpoint orientations, a significant interaction was found between condition and hand (*F*_3,33_ = 13.4, *p* < 0.001; see **Figure 4A**). *Post hoc* analysis testing the simple main effect of hand within condition revealed that rotation times were similar for the two hands in the L45R45 condition. When rotation magnitudes were 90^∘^ for the two hands (L90R90), rotation time was longer for the right hand (*p* = 0.047). Finally, when rotation magnitudes were different for the two hands, rotation time was longer for the hand rotating 90^∘^ (L45R90: *p* = 0.014; L90R45: *p* = 0.005).

For the incongruent endpoint orientations, a significant interaction between condition and hand (*F*_3,33_ = 53.93, *p* < 0.001) was also found. *Post hoc* analysis testing the simple main effects of hand within condition indicated that when rotation magnitudes were different, rotation time was longer for the hand rotating 90^∘^ (L45R90: *p* < 0.001; L90R45: *p* < 0.001; see **Figure 4B**). In contrast, when rotation magnitudes were the same for the two hands, rotation time was dependent on whether the two hands rotated 45 or 90^∘^. Rotation time was longer for the right hand in the L45R45 condition (*p* < 0.001), however, when the two hands rotated 90^∘^, rotation times were similar.

### RELATIVE DIFFERENCE BETWEEN RIGHT AND LEFT HANDS: TRANSPORT AND ROTATION

No significant main effects or interactions were found for the relative timing differences between the right and left hands at the start of transport. The mean difference between the hands was -7.0 ms ± 3.6 ms regardless of endpoint congruency or condition. In contrast, an interaction between endpoint congruency and condition (*F*_3,33_ = 9.0, *p* < 0.001) was found for the end of transport. The interaction between endpoint congruency and condition was further analyzed by comparing the effect of condition for the congruent and incongruent endpoints separately. The effect of condition failed to reach significance levels for the congruent endpoint orientations. For the incongruent endpoints a main effect of condition was found (*F*_3,33_=14.4, *p* < 0.001). *Post hoc* analysis using Fischer’s LSD test indicated that the L45R45 was significantly different than the L90R90 (*p* = 0.003) and the L90R45 (*p* = 0.002). Further, the L45R90 condition was significantly different than the L90R90 (*p* = 0.001) and L90R45 (*p* = 0.002) conditions. Specifically, the left hand ended transport before the right hand in conditions where the left hand rotated the object 45^∘^ (L45R45: relative difference = -72.85 ± 13.26 ms; L45R90: relative difference = -83.34 ± 23.85 ms). In contrast, the right hand ended transport before the left hand when the left hand rotated 90^∘^ (L90R90: relative difference = 45.56 ± 26.08 ms; L90R45: relative difference = 64.8 ± 33.1 ms).

For the start of rotation, an interaction between congruency and condition (*F*_3,33_ = 7.3, *p* = 0.001) was found. The interaction was further analyzed by separately comparing the effect of condition on the congruent and incongruent endpoints. The main effect of condition was significant for the start of rotation for the congruent endpoints (*F*_3,33_ = 15.82, *p* < 0.001). *Post hoc* analysis using Fischer’s LSD test indicated that the L45R45 condition was significantly different than the L90R90 (*p* = 0.019), the L45R90 (*p* = 0.031) and the L90R45 (*p* = 0.001) conditions. The L90R90 was significantly different than the L45R90 (*p* < 0.001) condition. Finally, the L45R90 and L90R45 conditions were significantly different (*p* < 0.001). Results indicated that the left hand began movement before the right hand when a 90^∘^ rotation of the left hand was required (L90R90: relative difference = -31.8 ± 14.4 ms; L90R45: relative difference = -68.2 ± 22.4 ms). In contrast, the right hand began rotating before the left hand when a 45^∘^ rotation of the left hand was required (L45R45: relative difference = 33.8 ± 21.4 ms; L45R90: relative difference = 74.95 ± 16.6 ms). The main effect of condition was also significant for the incongruent endpoints (*F*_3,33_ = 5.563, *p* = 0.003). *Post hoc* analysis using Fischer’s LSD test indicated that the L90R45 condition was significantly different than all other conditions (L45R45: *p* = 0.03; L90R90: *p* = 0.016; L45R90: *p* < 0.001). The left and right hands began movement approximately simultaneously for the L45R45 (Relative difference = -9.1 ± 16.9 ms), L90R90 (Relative difference = -8.11 ± 10.8 ms) and L45R90 (Relative difference = 3.57 ± 11.6 ms) conditions. In contrast, for the L90R45 condition, the left hand began movement 45.7 ± 10.8 ms before the right hand.

For the end of rotation an interaction between endpoint congruency and condition were found (*F*_3,33_ = 29.9, *p* < 0.001). The interaction was further analyzed by separately comparing the effect of condition on the congruent and incongruent endpoints. For the congruent endpoints, the main effect of condition was significant (*F*_3,33_ = 5.719, *p* = 0.003). *Post hoc* analysis using Fischer’s LSD test indicated that the relative timing for the end of rotation was significantly larger in the L90R90 (-87.7 ± 25.5 ms) condition than in the L45R90 (-28.9 ± 37.8 ms, *p* =0.009) and L90R45 (10.7 ± 27.5 ms, *p* = 0.001) conditions. The timing difference in the L45R45 was -47.7 ± 38.33 ms and did not differ significantly from any other condition. For the incongruent endpoints, a main effect of condition was also found for the relative timing at the end of rotation (*F*_3,33_ = 48.615, *p* < 0.001). *Post hoc* analysis using Fischer’s LSD test indicated that the relative timing for the end of rotation was significantly different in the L45R45 condition than in the L90R90 (*p* < 0.001) and the L90R45 (*p* < 0.001) conditions. Further, relative timing at the end of rotation was significantly L45R90 conditions than in the L90R90 (*p* < 0.001) and L90R45 (*p* < 0.001). Finally, the L90R90 and L90R45 conditions were also significantly different (*p* = 0.001). Specifically, the left hand ended rotation before the right hand in conditions where the left hand rotated 45^∘^ (L45R45: relative difference = -176.3 ± 37.6 ms; L45R90: relative difference = -209.5 ± 32.51 ms). In contrast, the right hand ended rotation before the left hand in conditions where the left hand rotated 90^∘^ (L90R90: relative difference = 91.2 ± 46.73; L90R45: relative difference = 212.3 ± 49.5 ms).

### RELATIVE DIFFERENCE BETWEEN TRANSPORT AND ROTATE COMPONENTS: CONCURRENCY

**FIGURE 5:**
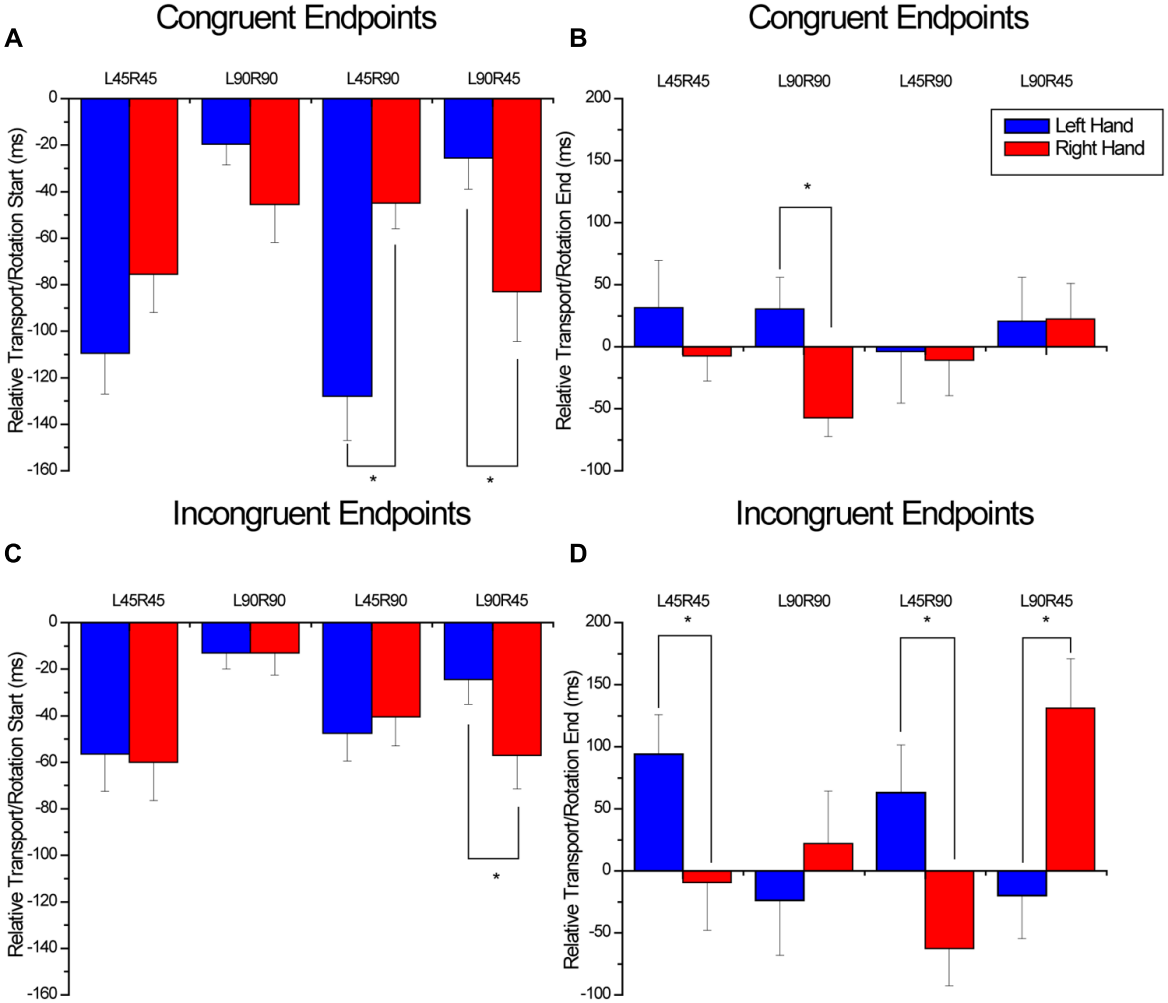
**Relative timing between the start of object transport and object rotation for the congruent **(A)** and incongruent **(C)** conditions as well as the relative timing between the end of transport and the end of rotation for the congruent **(B)** and incongruent **(D)** conditions.** For the x-axis titles, L and R refer to the left and right hands, and 45 and 90 refer to 45 and 90^∘^ rotations. Overall, these four figures demonstrate an inconsistent pattern of coordination between the transport and rotation components regardless of whether the endpoints were congruent or incongruent. *denotes significant (*p* < 0.05) differences between means and error bars represent SE.

A significant interaction between endpoint, hand, and condition was found for the relative time difference between the start of the transport and rotation components (*F*_3,33_ = 12.41, *p* < 0.001). This interaction was further decomposed by separately comparing hand × condition for the congruent and incongruent orientations. An interaction between condition and hand was found (*F*_3,33_ = 18.3, *p* < 0.001) for relative transport/rotation start in the congruent condition (see **Figure 5A**). Simple main effects analysis comparing the left and right hands within each condition indicated that when the hands rotated different magnitudes, the hand rotating 90^∘^ began the rotation component sooner than the hand rotating 45^∘^ (L45R90: *p* = 0.001; L90R45: *p* = 0.012). For the incongruent endpoint orientations, an interaction between condition and Hand was also found (*F*_3,33_ = 3.2, *p* = 0.037) for relative transport/rotation start (see **Figure 5C**). Simple main effects analysis comparing the left and right hands for each condition indicated that the only significant difference in relative timing between the two hands was for the L90R45 condition, where the left hand began rotating sooner than the right hand (*p* = 0.003).

For the end of the movement, an interaction was found between endpoint, hand and condition (*F*_3,33_ = 18.2, *p* < 0.001). This interaction was further decomposed by separately comparing Hand and condition for the congruent and incongruent orientations. For the congruent orientations, a significant interaction between hand and condition was found (*F*_3,33_ = 3.15, *p* = 0.038; see **Figure 5B**). Simple main effects analysis comparing the left and right hands for each condition indicated that the only significant difference in relative timing between the two hands was for the L90R90 condition (*p* = 0.02). Here, the left hand ended transport approximately 30 ms after the completion of the rotation component, whereas the right hand ended rotation 60 ms after the end of transport. For the incongruent condition, an interaction was found between condition and hand (*F*_3,33_=23.2, *p* < 0.001). As shown in **Figure 5D**, the relative time difference between the end of transport and end of rotation was different for the two hands for all conditions except the L90R90 condition (L45R45: *p* = 0.026; L45R90: *p* = 0.002; L90R45: *p* = 0.007).

## DISCUSSION

With the current study, we were interested in understanding how goal and movement congruency influenced performance in a bimanual transport, rotate, and place task that required precision at the endpoint. Previous work has indicated that for bimanual tasks, goal congruency (or end-state planning) can constrain movement planning and dominate over the motor actions necessary to achieve these goals (Kunde and Weigelt, 2005; Weigelt et al., 2006). In contrast, other work has shown that movement symmetry can dominate over goal symmetry in a task dependent way (van der Wel and Rosenbaum, 2010; Huhn et al., 2014). According to Huhn et al. (2014), these previous results suggest a flexible hierarchy, where multiple constraints can take precedence depending on the task. The purpose of the current study was to determine how this flexible hierarchy would extend to tasks with increased precision requirements. Further, it was unclear from previous work whether similar constraints influence each component of a movement in a similar fashion, or whether independent effects would be seen at the component level. We analyzed the kinematic performance of the transport and rotate components separately for tasks that resulted in congruent and incongruent end-goals. The separate analysis of these two components revealed differences in the way the end-goal and movement constraints influences the planning and performance of the task.

### OBJECT TRANSPORT

The results of the kinematic analysis of the object transport component revealed the strong influence of goal congruency on movement planning and execution for our task. In particular, when the required rotations for the two hands resulted in symmetric postures at the end-goal, movement times were similar for the two hands. Further relative timing differences between the hands at the start and end of movements were small (i.e., ∼6 ms) regardless of hand or condition. In contrast, for end-goals where rotations of the hands resulted in asymmetric postures, condition and hand interacted to influence movement time. These differences in movement times, which could be as large as 80 ms, resulted from synchronous start times but asynchronous end times for the two hands. These results are particularly striking when we consider the incongruent R45L45 and R90L90 conditions. In these conditions, the transport component and rotation distance remained the same for the two hands. Only the ending posture differed between the two movements. If movement symmetry was an important planning parameter in our bimanual transport and rotate task, we would have expected similar movement times and small movement asynchronies for the two hands. Thus, our results for the transport component replicate those of Kunde and Weigelt (2005) who suggested that planning and executing bimanual movements is determined by the congruency of the endpoint goal and not the coherence of the muscles used to reach the end goal.

Kunde and Weigelt (2005) proposed two potential mechanisms for the facilitatory effect of goal congruence. First, they suggested that congruent goals simplify the performance of bimanual actions because they do not require the maintenance of two separate goal postures. Second, based on the work of Diedrichsen et al. (2003), they suggested that incongruent movements require separate goals to be assigned to individual hands, which is a more difficult task than assigning the same goal to both hands. While we agree that these cognitive explanations likely account for some of the facilitatory effects of congruent goals, we would also like to suggest a third factor: sensory feedback. In particular, it has recently been shown by several research groups (including ours) that the requirement to obtain visual feedback from the two hands during the performance of slow, complex bimanual movements (i.e., reach to grasp, orientation tasks) can have a significant influence on the synchrony of movement performance (Mason and Bryden, 2007; Bingham et al., 2008; Mason and Bruyn, 2009; Srinivasan and Martin, 2010). This is in contrast to speeded, less complex aiming movements, like those used by Kelso (1995), where the fast transport times (∼300 ms) reduce the time available for saccadic monitoring of both hands, thus leading to movement synchrony. In tasks like the one used in the current experiment, participants must divide their visual fixations between the two separate target locations in order to successfully achieve the task goal. Consistent with the current results, Mason and Bruyn (2009) reported an increase in the number of overt shifts in visual attention during incongruent movements when compared with congruent movements. Further, the visual feedback must be integrated with the felt position of the limbs obtained via proprioceptive feedback (Jackson et al., 2000). When participants place targets in congruent end-goal orientations, the felt and seen orientations of the two limbs should be similar when the goal orientation is achieved. This expected similarity of the afferent sensory information about the two final hand postures may provide a referent to facilitate recognition of errors at the end goal position. In contrast, when the end-goals are incongruent, visual, and proprioceptive feedback from each limb is dissimilar, resulting in an increased processing load, and no between-limb referent for determining position errors. As such, the integration of visual and proprioceptive feedback may be facilitated in tasks that require congruent end-goals for the two hands. This is necessarily independent of the similarities between the movements required to reach the end goal.

### OBJECT ROTATION

In contrast to the clear determining influence of end-goal congruency on the temporal synchronization of the two limbs during object transport, end-goal appeared to play a smaller role in defining movement execution during object rotation. Even when the required rotations for the two hands resulted in symmetric postures at the end-goal, object rotation times were influenced by rotation magnitude and the hand performing the rotation (i.e., movement symmetry). Interestingly, when rotation magnitudes were similar for the two hands, rotation time was longer for the right hand than the left hand. Recently, Srinivasan and Martin (2012) reported that with practice, the left hand is prioritized as the primary hand and gaze is biased toward that hand during movement performance. Although participants in our study did not receive extended practice on our task, our results may indicate that the left hand was prioritized from the beginning due to the novelty of the task. This is supported by the results for relative timing between the hands. Specifically, the left hand began rotation prior to the right hand in half the conditions and ended prior to the right hand in all but one condition. Since our skill required precision at the endpoint, and our participants were all right-handed, they may have biased their fixations toward the left hand, only switching fixation to the right hand at the end of the movement. Thus rotations of the object in the right hand necessarily took longer to complete. Unfortunately we cannot definitively confirm this hypothesis since we did not measure eye movements. Additional work will need to be completed to determine whether prioritizing of the left hand was in fact a contributor to the asynchronies noted for rotation time.

Finally, we feel it is import to highlight the results of the analysis of concurrency of the transport and rotate components as a potential metric for inferring some of the planning processes that are employed as participants perform tasks with multiple components. **Figures 5A,B** illustrate the relative timing differences between the transport and rotate components for the congruent end-goals. **Figures 5C,D** represent the incongruent end-goals. What is interesting to note are the differences in the relative timings between the hands, particularly for the congruent conditions (see **Figures 5A,B**). These within condition/between hand differences highlight the fact that despite consistent hand transport performance, the performance of the object rotation component was highly asynchronous within the context of hand transport even with end-goal congruency. Specifically, note how the two hands start the rotation component at similar times with respect to the transport component for the congruent rotation conditions, but end the rotation component at completely different times. In fact, for the L90R90 condition, the left hand ends rotation prior to the end of transport, whereas the right hand ends after the end of transport. This suggests that at the planning level, end-goal congruency can be incorporated into the movement plan for the transport component, but for the rotation component, the plan must include necessary flexibility for the assessment of sensory feedback at the end of the task.

In sum, our results support the recent conclusions of Huhn et al. (2014) that constraints do not exert their influence on movement planning and performance in a winner take all fashion. Instead we have shown in the current work that they are integrated in a flexible fashion to exert differential influence on each component of the movement. In particular, we found that goal-congruency had a strong determining influence on the symmetry of hand transport to the target location. In contrast, the execution of the object rotation component was determined by a combination of end goal congruency and movement symmetry. The execution of each component may have also been influenced by the need to integrate visual and proprioceptive information for goal achievement.

## Conflict of Interest Statement

The authors declare that the research was conducted in the absence of any commercial or financial relationships that could be construed as a potential conflict of interest.

## Footnotes

1 This experiment is a follow-up to Mason and Bryden (2007) in which we used a blocked design but did not control for endpoint congruency. To avoid adding a confounding factor which would prevent us from comparing our results to our previous work, we chose to maintain the blocked trial order. Follow up studies should test random trial orders to see whether blocking versus randomizing has an effect.
